# Russia and the Medical Drug Trade in the Seventeenth Century

**DOI:** 10.1093/shm/hkw106

**Published:** 2016-11-16

**Authors:** Clare Griffin

**Keywords:** Russia, Early Modern, Drug Trade, Global History, European Medicine

## Abstract

This article deals with the trade in medicines into Russia in the seventeenth century. Both the early modern medical drug trade, and Russian medicine, have previously received substantial attention, but no work has thus far been undertaken on the Russian angle of the drug trade. Drawing on previously unused documents, this article traces the kinds of drugs acquired by the Moscow court. In contrast to the dominant view of official Russian medicine as divorced from native healing practices and fundamentally reliant upon Western European trends, these documents reveal that drugs were sourced as locally as Moscow markets, and from as far afield as East Asia and the Americas, but that not all drugs were accepted. As many of these imports came through Western European markets, this article also sheds further light on what drugs were available there, demonstrating the great diversity of drugs traded in early modern Europe.

Russia in the seventeenth century had a particularly unusual form of official medicine: until 1654, all medical practitioners employed at court or in the army were foreigners from Western Europe.^1^ Even after that date, Russians made up only a small proportion of medical practitioners until the late eighteenth century.^2^ Similarly, the majority of medical books available in Russia were imported from Western Europe, translated from Western European texts, or compiled from Western European sources according to Western European models.^3^ Other countries also brought in medical texts and practitioners from abroad, but to import so much of official medical practice was certainly atypical. Russia, then, seems a good candidate for a re-examination of the issue of early modern medical drugs, where the use of local versus foreign products has become a central question, as the whole official medical system until 1654 (and even after that) was arranged around importing medicine. It is perhaps surprising, therefore, that so little attention has been devoted to medical drug imports to Russia; although drugs have received peripheral attention in works focusing on other aspects of Russian medicine or trade, no one has ever used these records as a group to shed light on Russian involvement in the early modern medical drug trade.^4^

There are problems in tackling the Russian question. Very few customs records, so important to trade studies, are extant for Russia; not a single seventeenth-century custom book for the vital early modern Russian port of Arkhangelsk survives.^5^ Such documentary problems shift the study of the Russian medical drug trade in a specific direction, towards the records of the court medical department, the Apothecary Chancery. This department is central to Russian medicine of this period, and much has been written about its institutional history, and its medical practitioners, but not its medical drugs.^6^ Records for the Apothecary Chancery provide evidence regarding what was imported for official use at court. They also give some limited insight into the wider Russian market, as from at least the 1630s the Apothecary Chancery also supplied the army, from 1672 ran a shop selling medicines to Muscovites, and also purchased supplies from local markets throughout the century. In some ways these records follow what we already know about official Russian medicine: medical drugs were imported into Russia from the same ports, across the same period, and often in the very same ships, as foreign medical practitioners. Similarly, the volumes of drugs brought in officially, like the numbers of foreign practitioners, were sufficient for the court, but can only have provided a limited supply to the wider market. In other ways the picture of early modern Russian medicine provided by medical drug records is rather different: whereas practitioners came from a rather small group of European countries, drugs brought into Russia originated from as far afield as East Asia and the Americas. As the Russian court was acquiring a substantial proportion of its supplies from Western Europe, this then also provides an insight into the nature of medical drugs sales in Russia’s major trading partners, most notably Hamburg, Lübeck, Amsterdam and London. Work on the drug trade has often focused on the continuation of the East Asian spice trade and the importation of new American plants. Russian records strongly support the idea that both of these were major factors in seventeenth-century European medical drugs.

The records also show a greater degree of similarity between official and unofficial medicine than has otherwise been thought. Recent studies on unofficial medicine have focused on the practitioners, who were Russians, or non-Russian subjects of the Russian empire, and so unlike the foreign, courtly practitioners. These unofficial practitioners often got caught up in witchcraft trials over their practice, something that, despite persistent rumours of their magical practices, the court practitioners never endured.^7^ The private markets on which medicines were sold were investigated multiple times across the late seventeenth century, and then banned in 1701.^8^ The story of unofficial and official Russian medicine based on such material is one of difference and opposition. In stark contrast, medical drug records show the Apothecary Chancery buying supplies from these markets, and so demonstrate how similar the drug practices of official and unofficial Russian medicine may have been. Despite significant differences and opposition, there was common ground between official and unofficial Russian medicine, which can be uncovered through close examination of the drug records. Conversely, these documents also show a gap between Russian practice and its Western European sources: some drugs popular in Western Europe, notably theriac, were either heavily restricted, or banned outright, at the Moscow court. This article mines these almost untouched sources to revise our understanding of the links, connections and associations and, conversely, the conflicts, disconnections and oppositions, of official seventeenth-century Russian medicine with the wider world.

## Russia and the World, *circa* 1600

There is now a significant literature on the history of globalisation, global trade and global links in the early modern period.^9^ One central point that has emerged from these discussions is the uneven distribution of, and varied prices for, products around the early modern world. This idea has been discussed in the Russian context by Matthew Romaniello, with regards to Russia’s seventeenth-century ban on the importation of tobacco.^10^ Tobacco, a product consumed across the rest of the world, was banned in Russia for decades. Although this ban did not totally exclude tobacco from Russia—foreigners could import it for their own use, Siberian peoples were permitted to consume it, and there was rampant smuggling sponsored by the English government—it nevertheless meant a distinct restriction of the product’s circulation. Russia’s engagement with the medical drug trade, as with its engagement with tobacco, was an interaction with a global phenomenon fundamentally shaped by local considerations, in particular Russia’s experiences in the seventeenth century.

The Russian empire is an early modern phenomenon. As late as the fifteenth century, ‘Russia’ was a group of towns held together by a common language and culture, and by the filial relations of the princes of those towns. It was Ivan III (r. 1462–1505), in the very late fifteenth century, who brought the various towns under the suzerainty of Moscow, giving Russia its other early modern name of Muscovy. Muscovy then continued to expand as a power in the sixteenth century, with Ivan IV (r. 1547–84) conquering the khanates of Kazan’ (1552), Astrakhan’ (1556) and Sibir’ (1580). Following the death of Ivan IV’s son and successor, Fedor, in 1598, the country slid into a period of chaos known as the Time of Troubles, in which the realm was not entirely under the control of the centre. Having been elected to the throne in 1613, the new Romanov dynasty then went about consolidating the conquests of the sixteenth century, but added few of their own. Perhaps the most famous Romanov was Peter the Great (r. 1682–1725), under whose rule Russia adopted the term *Imperiia* (1721), and emerged as a world power. This startling trajectory—from near-collapse in the 1600s to a major role on the world stage in the 1700s—is one reason the seventeenth century has been named as a key, ‘transitional’ time for Russia.^11^

During the same period, Russia was developing its foreign links. Muscovy conquered many of its immediate neighbours to its East and South, and maintained some contact with the other Eurasian powers, in particular the Ottomans, Safavids and Qing. However, these links did not translate to a substantial presence of people from those empires at the Russian court. A substantial amount of time and effort was put into developing links with Western European powers. Three regions in particular developed relations with the Russian State: England, the German lands and the Netherlands.^12^ Partly this group of nations was determined by geography: all three groups had access to the North Sea and the Baltic, through which they could reach the Russian ports of St Nicholas, and later Arkhangelsk, in the White Sea, and the Baltic ports of Reval and Narva, through which goods and travellers could also reach Russia. Religion also played a role. The Russians, as Orthodox Christians, were suspicious of the Catholic Church, a suspicion that was compounded by their long-term rivalry with Catholic Poland-Lithuania. Notably, many of the European regions with which Russia maintained strong links were Protestant. In contrast to the limited number of people from Eurasian empires present in Moscow, there were increasingly substantial numbers of Western Europeans living and working there.

This was reflected in the kinds of foreign expert found in Moscow. The court recruited increasing numbers of such experts, in particular people skilled in medicine, warfare, firearms production, mining and construction. These were often recruited through diplomatic and trade links with Europe, and so many of these experts were English, Dutch and German Protestants, although other nationalities, and for that matter, some Catholics, were also present.^13^ Research by A. P. Oparina has shown that these men, for the most part, lived rather comfortable lives in Moscow’s foreign quarters, being well paid, having their own communities and churches, and even putting on plays in each others’ homes.^14^ Unlike most Russians, who rarely had links with foreign lands, these Moscow-based foreigners kept, and were officially encouraged to keep, links with their home governments and with other experts in their field based elsewhere in Europe. These personal links were as vital to shaping Russia’s imports of medicines as high-level diplomatic contacts.

## Russian Medicine

The importance of these diplomatic, mercantile and personal links are immediately obvious in the practitioners of official Russian medicine. From the 1480s on, the Russian court preferred to employ Western European medical practitioners over native healers. By the seventeenth century, the activities of foreign medical practitioners at the Russian court had been formalised as a part of the department known as the Apothecary Chancery, which existed until 1714, when it was replaced by the Medical Chancellery.^15^ The educational programme started in 1654 did train Russians to act as official medical practitioners, but only as apothecaries and surgeons, not physicians.^16^ In 1701, Peter the Great established legislation for private apothecaries to be licensed by the state for the first time, and the first two men to be so licensed were foreign medical practitioners, who also worked for the Apothecary Chancery.^17^ Sabine Dumschat provides statistics as to the origins of these foreign medical practitioners: Poland provided a number of surgeons, but most physicians and other medical practitioners were from England, the Netherlands or the German lands, Russia’s key Western European contacts.^18^ It was these foreign medical practitioners who provided the crucial link for the Russian court’s acquisition of foreign *materia medica.*

Whilst the kinds of medical practitioner being used remained broadly stable across the seventeenth century, the Apothecary Chancery’s list of patients fluctuated. The tsar’s family and high-level courtiers always had access to such services, apart from cases when nobles who had fallen out of favour were temporarily banned from using the department.^19^ From at least the 1630s, army servitors could also petition for treatment, and field surgeons were sent out from the department to be deployed with army regiments.^20^ In 1672, the department began to sell medicines to all Muscovites, with provisions made for those who wanted medicines but could not afford them.^21^ There were also some efforts to expand official medicine outside the capital, with attempts to set up pharmacies in Vologda and Kazan.^22^ By the late seventeenth century, centrally located Muscovites, and Russians in service to the army, had at least the possibility of accessing official Russian medicine.

This official medicine has left us a wealth of documents. Much work has already been done on the materials pertaining to the medical staff of the department, and to its institutional history.^23^ However, large numbers of documents remained virtually unused, in particular those relating to medical drugs. In large part due to fears of poisoning, the acquisition, storage and usage of consumable medicines were carefully tracked and recorded. These documents are extremely valuable to historians of the medical drug trade: trade records often gloss over medical products, whether medicines, equipment or books, subsuming them under general categories, whereas Russian court documents on medicine do the exact opposite, tracking every ounce of every substance brought into the department, where it was kept, how it was prepared and by whom, and to whom the final product was given. Moreover, these records also give insight into which medicines were acceptable or unacceptable: all medical practitioners swore an oath promising faithful service, an oath which details precisely what they could, or could not, do. Russian official medical records thus give a vital and fascinating insight into early modern medical drugs.

Despite the importance of official medicine, and the great interest of documents coming from official sources, we should remember that official medicine was also limited. For people in the provinces in particular it was almost completely out of reach. Given the size of the department, it is also unlikely that it could have even completely provided for the needs of Moscow. We also know that even those Russians who had access to official Russian medicine did not completely abandon local practices: Boris Ivanovich Morozov, a former head of the Apothecary Chancery, simultaneously consulted both an unofficial practitioner in his own employ and the Apothecary Chancery physician Samuel Collins during his final illness in 1662.^24^ Apothecary Chancery documents show the existence of a number of private traders on markets, in particular in Moscow, Arkhangelsk and the Western border town of Mogilev, selling either raw ingredients which could be made into medicines, or indeed the medicines themselves.^25^ Unofficial medicine was a huge part of Russian healing practices, apparently taking on greater significance in provincial centres, and, most likely, rural areas, far from the Apothecary Chancery’s ambit. Indeed, Andreas Renner has argued that, although historians regularly focus on official medicine, with unofficial medicine seen as ‘marginal’, in reality it was the other way round. Unofficial medicine was the norm, an almost totally unchallenged dominant force in how Russians healed themselves. Official medicine, in both the seventeenth and the eighteenth centuries, could not hope to compete.^26^

One problem for historians has been how to access this unofficial medicine. Practitioners were commonly not literate, and even if they were, they did not keep records. Almost the only way Russian unofficial healers enter the historical record is in trials, a situation in which we necessarily only get a partial, skewed and fundamentally negative picture of their activities. Nevertheless, Eve Levin has made great use of such documents to open up what we can know about unofficial Russian healing of the seventeenth century.^27^ An almost unexplored set of documents relating to unofficial practice comes from the Apothecary Chancery. As that department sourced some of its raw ingredients for medicines locally, from markets both in Moscow and further afield in the empire, Apothecary Chancery purchase lists can also tell us about unofficial medicine. In considering the Russian drug trade from this source, we can draw conclusions not only about the nature of medicine at court, but also regarding the nature of unofficial medicine, and how it may have co-existed with official medicine.

## Local and Imperial Drugs

Many studies have been written on Russia’s early modern empire, focusing in particular on the organisational, administrative and political questions of ruling such a large, culturally diverse polity.^28^ Less has been written about the uses of natural objects from within the empire, either by the court or by ordinary Russians.^29^ Studies focused on other European empires have shown how the use of local natural objects was of continued importance in the early modern period, despite the increased possibilities for importing natural objects from much further afield. Indeed, Alix Cooper’s work *Inventing the Indigenous* emphasises how the introduction of New World products into Central Europe in fact led to many intellectuals refocusing their interests on the value of local products.^30^ Materials from the Apothecary Chancery allow us to explore the potential medical uses of natural objects from the Russian empire, both at court and outside it.

Local markets certainly stocked local drugs. Apothecary Chancery records show the department purchasing a number of items which could have been sourced locally. In 1651, dill and juniper berries were amongst the supplies acquired from various Moscow markets.^31^ In 1654, the department purchased mint oil and wormwood oil from Mogilev.^32^ They were also able to source rosemary and basil (Arkhangelsk annual fair, 1672) and lavender and rosemary (Moscow markets, 1694).^33^ These objects were all common to both Russia and Europe, but there is reason to believe that at least a proportion would have been sourced from within the empire. One market trader, Vasilii Kirilov, questioned about his trading activities in 1699, claimed that he stocked his stall from items brought to him by rural people who collected plants in the fields near where they lived.^34^ The market trade in medical drugs, both within and outside Moscow, was at least partly based on locally-grown supplies.

Market traders provided their wares to the Apothecary Chancery, and also to unofficial healers. The unofficial healer who treated Boris Ivanovich Morozov in 1662 was certainly using local supplies, specifically a herb known as the *hare’s hoof* [*zaiach’e kopyto*].^35^ As discussed above, unofficial healers often came under suspicion of witchcraft; indeed, a substantial proportion of seventeenth-century witchcraft trials involve herbal healing.^36^ Although not all the substances presented as evidence in those trials were identified in the records, those that were, were often local in origin. In a case from 1628 the accused, Andrei Loptunov, was found carrying a root identified as Gooseflesh, given to him by an unofficial healer.^37^ On one occasion, in 1657, the defendant, Andrei Durbenev, stated that his materials grew locally:

One root is taken by people for stomach complaints [lit. womb] and for difficulty breathing, and the second root is for horses, it is given to broken-winded horses, and the third root is for teeth, it grows in fields and kitchen gardens.^38^

The Apothecary Chancery staff, to whom such evidence was commonly sent to gain their opinion, also sometimes made a statement on the origin of the herbs in question, as in 1664:

[there are] the herb *karniana*, another herb *kanisa*, and they [the Apothecary Chancery staff] said that those herbs are wild herbs [lit. field herbs].^39^

The use of the term ‘wild herbs’ or ‘field herbs’ partly denotes an uncultivated plant, but may also indicate that the herbs were to be found locally, in the fields. Like the market traders in medicines, unofficial healers also commonly relied upon local substances in their medicines.

Of all groups in Russia, the court had the greatest possibility of exploiting foreign sources for medicines. However, records show that the court invested substantial time and money in gathering at least a proportion of their medical drugs from around the empire. Such efforts were coordinated by the Apothecary Chancery, but necessitated the involvement of governmental couriers, governors, regional administrators and peasant populations both in regions close to Moscow and much further afield. Moreover, this process continued from the 1630s well into the eighteenth century.

Perhaps the most commonly collected objects from around the Russian empire were juniper berries, which were required in large quantities to be made into the alcohols in which early modern medicines were commonly suspended for convenient consumption. Rachel Koroloff has argued that collections of juniper berries were particularly centred on the vicinity of Iarolslavl’, an historically important town located 250 kilometers to the north-east of Moscow.^40^ Iaroslavl’s location is significant: the town is well within the borders of the historical Rus’ lands, making collections there very much an affair of Russia proper, and not the empire. Koroloff has shown how juniper berries were collected by local peasants as an obligatory service to the tsar [*povinnost’*], and then passed on to the Apothecary Chancery. Juniper berry collection, then, was an activity of the central Russian provinces, and one that was organised as a form of traditional service obligation.

From at least the middle of the seventeenth century, the Russian state also exploited the botanical possibilities of its wider empire, most notably Siberia. The use of Siberian natural riches was not new: for centuries, Siberian furs had been prized across Europe and the Near East.^41^ Koroloff has argued that Siberia, notably the area around the key administrative centre of Tobolsk, was exploited for its supplies of St John’s Wort.^42^ When in need of this item, the Apothecary Chancery would write to the governor of Siberia at Tobolsk, and instruct him to send suitably knowledgeable men into the fields and forests in search of this object. As with Juniper berries, the St John’s Wort would then be sent back to Moscow for use by the Apothecary Chancery. There was no obligatory collection of St John’s Wort akin to the Juniper berry *povinnost’.* Despite the differences in organisation, the collection of St John’s Root from the Tobolsk area was markedly similar to the collection of Juniper berries in the Iaroslavl’ region, including the central role of the ordinary apparatus of the state to undertake the process.

In only one instance did the seventeenth-century Russian state undertake a botanical expedition proper: the search for a Siberian source for rhubarb. Rhubarb had been traded using land routes across Eurasia—originating in the Chinese territories, and moved by Bukharan merchants to Russia, whence it was then sent on to Western Europe—since at least the middle of the sixteenth century.^43^ Due to the high demand for rhubarb in Western Europe, the Russian state had a stake in finding a domestic source for it. As Erika Monahan has shown, there was a short-lived attempt to collect rhubarb in Siberia, rather than import it from the Chinese, likely to have been motivated by commercial aims. However, as demonstrated by Monahan, these expeditions were exclusively organised by the state, and indeed documents pertaining to the expeditions were labelled *gosudareva dela* [lit. the Tsar’s business]—a state secret.^44^ In the case of the short-lived rhubarb expeditions, commerce certainly played a role, but only as a part of the state’s motivations, as it was still the state that controlled, organised and profited from such schemes.

Consideration of local drugs thus breaks down some of the barriers thought to have existed between official and unofficial medical practice in seventeenth-century Russia. Market traders, unofficial healers and the court medical department, all had an interest in using local drugs. The court did so on a huge scale, constructing, or attempting to construct, entire supply systems to funnel local and imperial medicines to Moscow. Private trade seems to have been much more small-scale, with individual market traders dealing one-on-one with certain peasant collectors. In the case of unofficial healers, they also seem to have been involved in rather small-scale dealings. It is also interesting that, despite the often negative attitude of Russian authorities towards unofficial medicine, they themselves used private market traders to source certain items. Although in many ways official and unofficial Russian medicine were very different, and sometimes in conflict, they both shared a common interest in local drugs, albeit sourced on radically different scales.

## The European Connection

Russia’s closest contacts—in terms of medicine, but also trade and diplomacy—in the seventeenth century were with a small group of Western European nations, most notably England, the Netherlands and the German lands. Earlier work on the Apothecary Chancery has established the fundamental influence of these groups of foreigners on the personnel, library and medical practices of the Apothecary Chancery. Less is known about the importance of the Western European connection for medical drugs. Apothecary Chancery records allow us to examine both the ports through which Russia sourced its medical drugs, and the geographic origin of those drugs, to assess the relative importance of Western Europe in that process. Conversely, the great detail of Russian records concerning this trade with major European markets also allows us to explore further the history of medical drugs in Western Europe.

Although Russian market traders sourced a proportion of their goods locally, some of those drugs may have been imported from Western Europe. In 1654, the Apothecary Chancery purchased mint oil and wormwood oil from Mogilev.^45^ They were also able to source rosemary and basil (Arkhangelsk annual fair, 1672), and lavender and rosemary (Moscow markets, 1694).^46^ They purchased senna from Mogilev in 1654.^47^ Such goods could have been either sourced locally or imported from Western Europe, where they also grew. The case of senna is significant. Senna is found in various regions, but the Russian term—*Aleksandreiskii list*—suggests a link with Alexandrine senna, which grows in Egypt but was commonly traded in Europe. This is not surprising: Mogilev was a border town, meaning local merchants had particularly good access to foreign goods. The presence of senna demonstrates that some imported medicaments were being traded on Russian markets. Despite the problems of identifying the specific geographic origin of Eurasian plants, it does seem that unofficial Russian medicine had some access to medical drugs from Western Europe.

The Russian court provides much clearer evidence for the role of Western Europe in medical drug imports, as import lists from the Apothecary Chancery tell us not only what was imported, but from where. Many of the items included in these shipments could be Western European in origin: rosemary, lavender and mint, all repeatedly appear in such lists of imported drugs. However, again we face the issue of not knowing exactly where such plants originated, as they grew in so many locations. Given the central importance of Western Europe for Russian medicine overall, it is also worth considering the proportion of imports that can be more or less conclusively traced as being from Western Europe. In the earliest available list, from 1602, drugs which were European or Mediterranean in origin represent two-thirds of the 164 drugs.^48^ In the 1645 import list of items from Hamburg, objects of European origin take up perhaps half of the 176 items listed.^49^ A similar proportion of the 1694 import was European.^50^ Western European plants did play a very substantial role in Russian medicine, but they by no means occupied the dominant position that Western European medical practitioners, or Western European texts, did.

Import lists also provide valuable information regarding the ports of origin for these shipments. Drug imports were often arranged by medical staff, who arrived at court with such supplies, such as in 1602, when the English apothecary James Frencham arrived with medical drugs (in unknown quantities) for the court.^51^ As well as bringing drugs with them on arrival, medical practitioners already at the Russian court had shipments sent to them, usually via Arkhangelsk: the English physician Arthur Dee claimed to be receiving annual deliveries of goods through that port during his stay in Russia in 1621–34.^52^ Little is known about these shipments other than their existence (as the physicians in question had to petition for sledges to be sent to Arkhangelsk to collect the goods), but the personal nature of the shipment would seem to suggest relatively small quantities being sourced from the medical practitioners’ home country. Early seventeenth-century imports were arranged by medical practitioners themselves, and seem to have occurred on arrival, and periodically thereafter.

From the 1640s on, drugs were not only procured via medical practitioners working at the court, but also via merchants. Even in these cases, medical practitioners continued to play a significant role: they drew up ‘shopping lists’ of items the merchants were to source for the court.^53^ The earliest extant record of such an import dates from 1645, and records 176 different types of drugs being brought to the Apothecary Chancery from Hamburg.^54^ Hamburg is the most frequent port of origin for the extant records for such merchant-supplied drug imports from Europe: of 13 such imports that can be definitively traced in the Apothecary Chancery records, nine were from that city. The other ports were Amsterdam, which supplied two such deliveries, and London, for which there is one listing. These figures should be approached with caution: the Apothecary Chancery records are not complete, and the importance of Amsterdam and London is likely to be underrepresented. Nevertheless, the high number of extant documents mentioning Hamburg is significant, as it somewhat contradicts other trade data: Hamburg was certainly a significant actor in seventeenth-century Russian trade, but Lübeck was the most important of the Hansa towns, and the Dutch dominated overall in Russian foreign trade in the latter seventeenth century.^55^ However, the German lands were in the ascendancy in Russian medicine in this period: between 1660 and 1696 the number of German medical practitioners at the Russian court more than tripled (going from 8 to 37), whereas the number of English practitioners halved (from 6 to 3), and the number of Dutchmen remained stable (5).^56^ Across the seventeenth century, the drug imports of the Apothecary Chancery grew substantially, with increasingly large shipments being brought in by merchants supplementing the continued use of medical practitioners to source drugs. However, the structure of that trade was reliant upon the structure of Russian medicine, and not Russia’s foreign trade.

The role of European substances in official and unofficial medicine was broadly similar to that of local and imperial drugs. Both courtly and non-courtly practitioners made use of Western European contacts to source medicines, with significant overlap as to what specific items were sourced. The main difference seems to have been one of scale. The court had the resources and connections to bring in shipments of several hundred items, and did so periodically from the 1640s on. From what we know of unofficial medicine, private traders were not able to bring in such huge quantities. The difference between court and non-court usage of European drugs was one of scale, not a fundamental rift.

## The Far East and the Spice Trade

A central question of the early modern drug trade has become how the shifts in trade networks, including the more frequent travel to East Asia, affected the medical drugs available in Europe. This can partly be addressed by considering the long-term history of the spice trade, as spices were often also medical drugs. Indeed, Timothy Walker and Harold Cook have argued that, in some senses, ‘medicines were a subcategory of the spice trade’.^57^ Patrick Wallis’ work on the London Port records has shown that the spice trade, and its double, the medicines trade, grew substantially, in particular during the seventeenth century.^58^ Russian court records, listing as they do even small quantities, are therefore a good test case for the fluctuations of this group of drugs across the seventeenth century in a different setting.

Spices were commonly found on Russian markets in the seventeenth century. In 1654, the Apothecary Chancery was able to buy seven different spices from markets in the border trade town of Mogilev, including nutmeg, saffron and cinnamon.^59^ In 1662, the department sourced cloves and other unspecified spices [*priannye zel’i*] from the annual fair at Arkhangelsk.^60^ The Moscow markets also carried various eastern goods: the department brought camphor (1651, 1684 and 1685), nutmeg (1687) and cinnamon and saffron (1691) from there.^61^ A collection of receipts from 1694 lists nutmeg, mace, cinnamon, camphor, sandalwood, and saffron all being purchased from the Moscow markets.^62^ Although these records most directly speak to the Apothecary Chancery’s use of spices as medicines, these markets did also supply other practitioners, and so five or six different spices were certainly available for use by unofficial medical practitioners in Muscovy.

Seventeenth-century Russian documents also provide ample evidence of the continuous use of various spices as medicines at the court across the century. All extant seventeenth-century import lists, dating from 1645 through to 1696, list multiple spices, notably cinnamon, saffron, cardamom and sandalwood. Mace, china root and pepper also frequently appear. Two mid-century stock-takes of Apothecary Chancery supplies also list all these items.^63^ The spices listed include all those available on the market, and a number of other spices apparently less common on the markets, such as china root and pepper. Across the seventeenth century, spices were regularly procured by the Russian court for use in official Russian medicine, in a somewhat greater variety of types than was commonly available outside the court.

The shift in usage of these goods across the century is better explored through a limited number of items. Nutmeg and cinnamon are two of the most commonly encountered spices in Russian drug documents. Tracing their frequency, and the quantities in which they were imported, tells us more about how spices as medicines evolved across this period. There are limitations to what can be known from the absolute quantity of a transported good, as the amount needed for a dose varied from ingredient to ingredient, and from recipe to recipe, and may also have varied across time and between different medical practitioners; moreover, the Russian documents only provide information on the quantities of drugs from the 1640s, and in some cases the 1660s, onwards. However, looking at the traded quantities does allow us some idea of how important a drug was likely to have been, and also how its importance may have changed over time. This is demonstrated by the changes in the quantities of nutmeg available between the 1660s and the 1690s. In the 1660s, nutmeg was traded in quantities of 3 *funty* (1.2 kg).^64^ By the 1680s and 1690s, this had increased to 20 *funty* (8.2 kg), although only in some shipments; in others, the quantities remained similar to what they had been in the 1660s.^65^ Nutmeg was thus imported consistently, and that trade neither collapsed, nor substantially increased, between the 1660s and the 1690s.

Cinnamon provides a rather different picture. From the 1640s to the 1670s, cinnamon was traded in *zolotniki*, a contemporary Russian measure of 4.226 grams, commonly used to measure small amounts of high-value items, such as precious metals.^66^ By the 1690s, it was being traded in *funty*.^67^ One *funt* was equivalent to 96 *zolotniki*, and so this alone signifies a substantial upward trend in the quantities traded.^68^ Moreover, cinnamon was traded in quantities of up to 100 *funty*, a huge increase from the earliest recorded trade of 16 *zolotniki*. In today’s measurements, in 1646 cinnamon was being traded in quantities of around 65 grams, but in the 1690s, it was traded in quantities as large as 40 kilograms.^69^ Quantities of cinnamon, then, apparently increased rather substantially across the seventeenth century.

Spices as medical drugs thus continued to be important in early modern Russia. Indeed, there is even some evidence that the quantities of certain spices being imported increased rather substantially across the century. Spices were available in large quantities, and indeed some variety, even in unofficial medicine; the availability at court was greater, both in quantity and type, as seems to be the case with all drugs. The Russian evidence thus broadly mirrors that of Wallis’s data for London, showing the continued significance of spices as medicines across the seventeenth century.^70^

## The Americas and the Problem of New Drugs

The other substantial shift in early modern trade networks with potential repercussions for medical drugs was the discovery and exploitation of the New World by European powers. Certainly Spain and Portugal, two of the early major European presences in the Americas, invested much time and effort into trying to commoditise the medicinal plants of their new colonies. However, the extent to which they were successful is debated. Following the scepticism of a number of scholars as to the uptake of American drugs in Europe, Valeria Finucci has written that, ‘[i]n the end, historically speaking, and no matter the particular interests of merchants and the experimentations of doctors and apothecaries, the New World therapeutics did not have much of an impact on the official pharmacopoeia, which kept its fairly traditional approach well into the eighteenth century’.^71^Other scholars have challenged this, notably Teresa Huguet-Termes and Wallis, both of whom argue for a significant role for American drugs, certainly by the mid-seventeenth century.^72^ Seventeenth-century Russia had no American colonies, and no direct trade links to Spain or Portugal.^73^ Nevertheless, several American drugs made their way to Moscow. The presence of American drugs in Russia is thus of particular interest, as it involved moving the products first into and then across Europe, and so points towards a regular early modern trade in those drugs in Western Europe as well.

The shape of the trade in American drugs is rather different to that of spices. American drugs were available in Russia outside the court, but not until rather late. In 1694, the Apothecary Chancery procured sassafras and cinchona, two of the most important American drugs, from one market seller [*torgovii chelovek*] Ivan Efimov, who worked on the Vegetable market in Kitai-gorod.^74^ This is the only record of the department obtaining American drugs from Russian markets. Although this evidence speaks to the purchasing of American drugs by the court, the fact that a privately trading merchant would have American drugs in stock suggests that they also had a market outside the court, amongst ordinary Muscovites. In contrast to the significant variety of different spices available on various market stalls both within and outside Moscow from at least the mid-seventeenth century on, the variety of American drugs available in that trade seems to have been much more limited, and to have come about much later.

More evidence comes from the court. American drugs appear there as early as 1602, as the drugs imported by Frencham included an unknown quantity of sassafras.^75^ This plant was particularly found in the Spanish colonies, known as New Spain, and its use was popularised by Monardes. The periodicity of its spread around Europe is debated, but English apothecaries had it in their inventories from at least the 1590s.^76^ American drugs then began to regularly appear in the Russian records from the late 1640s. Four American drugs appeared multiple times in the import records: alongside sassafras, we also find cinchona, sarsaparilla and guaiacum. Sassafras was one of the most consistently traded objects, appearing in import records eight times between 1645 and 1694.^77^ In 1660, cinchona appeared in the Russian import records for the first time, and was traded twice more, in 1667 and 1694.^78^ Cinchona, later known as quinine, but often referred to in early modern Europe as Jesuits’ bark or Peruvian bark, was sourced across South America, including Portuguese-controlled Brazil and, most famously, Spanish-controlled Peru. It was introduced to Europe as a medicine in the 1640s, in particular being used as an anti-febrile.^79^ Sarsaparilla appeared in Russian records in 1667, and again in 1694.^80^ Jesuit missionaries played a significant role in bringing this plant from its native ground of Brazil to Portugal sometime in the late sixteenth or early seventeenth century.^81^ It was particularly valued in treating syphilis, and in purifying the blood. Last to appear in the Russian import documents was guaiacum, or lignum vitae (1683 and 1692), sourced from New Spain and available in Europe from the middle of the seventeenth century.^82^ At least one American drug was certainly available at the Russian court from the 1600s, and several different American drugs were regularly available at court during the second half of the seventeenth century.

These documents also tell us something about the changing quantities of American drugs being traded across the late seventeenth century. Sassafras, the earliest drug to be imported, was sourced in fairly significant quantities from the mid-seventeenth century: 1 *pud*, or 40 *funty* (16.38 kg) in 1646.^83^ This is significantly larger than the mid-century import totals for certain spices, some of which were traded in fractions of a *funt,* not multiples of it (see above). The shipment of 1667–68 was a mass shipment, and also contained huge quantities of American drugs, the largest being the 220 *funt* 4 *zolotniki* of sassafras, equivalent to more than 90 kilograms, one of the largest quantities of an individual drug ever imported by the Russian court.^84^ Later shipments did not contain such huge quantities, with only 1 *funt* being imported in 1692. However, this cannot be taken as straightforward evidence of a decline in the importation of American drugs. In 1692–94, sassafras, cinchona, sarsaparilla and guaiacum were all imported in some quantity, along with 50 *funt* (20 kg) of sassafras.^85^ These records show a consistent demand for substantial quantities of American drugs by the Russian court across the late seventeenth century.

As well as their importance for the history of Russian medicine, these shipments are also significant for understanding the trade in American drugs outside Russia. On multiple occasions from 1645, American drugs were included in general shipments procured from European markets. This was not always the way medicines were procured by the Russian court: unicorn horns, mostly narwhal tusks, but commonly traded—for large sums of money—as the horn of the mythical land unicorn, were only ever acquired through special commissions.^86^ American drugs, then, were more accessible in Europe than unicorn horns. It should be remembered that the Russian court relied on very serious trade centres: London, Amsterdam, Lübeck and Hamburg, places with huge trade turnovers and excellent international connections. Nevertheless, the fact that 90 kilograms of sassafras could be sourced from Hamburg in the 1660s without, apparently, any special arrangements being made, points to American drugs being a fairly normal part of the medicines trade in Europe at that point. This undermines the stance of Finnucci and others, who claim that American drugs were not a serious part of early modern European medicine.^87^ Rather, such large quantities of American drugs being available suggests that they were a commonly traded medicine by the mid-seventeenth century, supporting the arguments of Wallis and Huguet-Termes.^88^ On the evidence of the Russian documents, there was a fairly stable trade in American drugs within Continental Europe by the middle of the seventeenth century.

Fewer American drugs appeared in Russia than spices, and those that were present had less of an impact outside court. Nevertheless, they were a significant part of early modern Russian medicine. American drugs reached unofficial medicine by the 1690s, nearly a century after they first appeared in Russian court records, in 1602. Although there were fewer kinds of American drugs being traded, those that were traded were exchanged in substantial quantities, likely speaking to a certain level of popularity. These huge quantities also tell us about Russia’s suppliers. There seems to have been a relatively stable, if not mass, trade in American drugs in Continental Europe by at least the 1660s. American drugs, despite the doubts of certain scholars, were a regular part of the pan-European drugs trade by the mid-seventeenth century.

## Dangerous Drugs? Animal Parts and Corpse Medicine

Early modern official Russian medical records show substantial appropriations from Western Europe. It is important also to note where Russian practice not only diverged from Western European norms, but when those norms were explicitly rejected. The selective nature of early modern Russian medicine has been discussed by Eve Levin and Andreas Renner, both of whom have emphasised the fact that Russians chose to import Western European medicine, it was not forced upon them.^89^ Such discussions fit into a broader trend within the global history of medicine, tracing how local factors shaped the reception of global trends. Londa Schiebinger has argued that objects that are not assimilated tell us as much about the drug trade as those that were, highlighting the example of a West Indian abortifacient popular in its region of origin but almost unknown in Europe despite significant contemporary demand for such exotics.^90^ What do Russian rejections of certain medical drugs widely used in Western Europe, within the context of a broader acceptance of that tradition by Russians, tell us?

Although it was the Western European medical practitioners employed at court who compiled lists of medicines to be purchased, Russians themselves controlled what was used as a medicine in the Apothecary Chancery. Like all Muscovite servitors, the medical staff of the Apothecary Chancery swore a service oath. Such oaths included a promise of service and obedience to the tsar, and also more or less detailed lists of essential or forbidden behaviour.^91^ In the case of the Apothecary Chancery, these oaths specify aspects of medical practice that are approved of or banned.^92^ In an oath dating from the early 1630s, Apothecary Chancery medical staff had to swear not to prescribe ‘unclean mummia nor any evil snake poison nor other poisonous animal nor reptile nor bird [nor] any evil and unclean compound that could harm health’.^93^ The reference to mummia is clear and unequivocal: Apothecary Chancery physicians are banned from using this substance. Mummia refers to a powder made from embalmed corpses, originally ancient Egyptian mummies, which was commonly used in medieval and early modern Europe, and one of a group of drugs known as ‘corpse medicine’.^94^ In the sixteenth century, medical humanists began to trace the evolution of the concept of ‘mummia’, and sparked a learned debate over its origins and usage, leading to its eventual decline in the opinions of medical men. Popular practice did not follow so easily, and mummia remained in use well into the eighteenth century.^95^ It is thus strange that the Russian court would exclude such an important European medicine from their practice. Moreover, the adjective used to describe mummia—*nechistii*—means unclean in a ritual sense, or even evil. In the first half of the seventeenth century, mummia was thus banned from use at the Russian court, with the Russians choosing to exclude one of the most powerful medicaments of the Western European pharmacopoeia from their practice.

The references to animal-based medicines in the oath are less clear. The oath explicitly rejects only one group of animals from use in medicine—poisonous ones—but the text does not list exactly which animals that would mean. The term I here translate as reptile—*gad*—can also mean something akin to vermin, a term of abuse as well as a zoological descriptor. The phrase ‘evil snake poison’ is also vague, but significant. The key ingredient in one of the strongest and most popular early modern medicines, theriac—a panacaea—was viper flesh, then thought to be poisonous.^96^ Moreover, another common ingredient of theriac was mummia.^97^ The common association of theriac and mummia, and the association here in the text between an ‘evil snake poison’ and mummia, would seem to suggest that the ‘evil snake poison’ was indeed theriac. This interpretation would also fit well with the remainder of the phrase, in which all medicines containing parts of poisonous animals are banned, as theriac would in any case fit into such a category. Moreover, in *The Present State of Russia*, the English Physician Samuel Collins, who had worked at the Russian court in the 1660s, specifically notes that ‘theriaca’ is banned in Russia ‘because it has Vipers flesh in it’.^98^ Thus, at least in the 1630s–1660s, the Russian court banned corpse medicine and seriously restricted the use of animal-based medicine, in the process excluding two of the most potent items of the Western European pharmacopoeia—mummia and theriac—from their practice.

The prohibitions on prescribing animal-based medicines seem to have been enforced. As well as his comments about theriac, Collins also complained that he was not permitted to prescribe medicines containing animal parts during fast periods, as it conflicted with the prohibitions of Russian Orthodoxy, writing ‘If a Medicine has *Cor. cervi* [deer horn], *ungul*. *Al.* [moose hoof] or *pil. lepor.* [hares’ hair] in it, they will not take it, though to save their lives, so precise are they in observing their Fasts’.^99^ However, several of Collins’ prescriptions have survived, showing that he commonly prescribed crabs’ eyes to patients.^100^ The Apothecary Chancery thus instituted a restriction on the use of animal-based medicines, not an absolute ban.

Import records also support the idea of the partial use of animal-based medicines at the Russian court. Certain animal-based medicines were imported, but the variety is limited, with only four items regularly appearing: crabs’ eyes, deer horn, scorpion oil and spermaceti. However, the quantities in which they were imported were substantial. Particularly in the second half of the seventeenth century, all items were imported regularly, in multiple *funty*, sometimes over 100 *funty* (40 kg) at a time. It is also significant that animal medicines appear only sporadically in the trade documents from the first half of the seventeenth century, and in every shipment in the second half of the century. No animal medicines were imported in 1602, and only spermaceti was imported in 1645.^101^ In contrast, shipments from the 1670s onwards feature all four medicaments, and in 1692 5 *funty* of scorpion oil, 8 *funty* of spermaceti, 20 *funty* of crabs’ eyes and 120 *funty* of deer horn were imported.^102^ This might indicate a growing acceptance of animal parts in medicines in the second half of the seventeenth century.

This change in the status of animal-based medicines in the late seventeenth century may be linked to two reports written for the Apothecary Chancery in the 1660s. A number of animal-based medical drugs are discussed in a 1664 report written by Dr Andreas Engelhardt. Interestingly, this document does not mention any of the most commonly recorded animal parts from the trade documents, nor, for that matter, theriac or mummia. Instead, the report focuses on parts of other animals: bears, wolves, foxes and hares.^103^ These animals were much more readily available than, say, spermaceti; bears were regularly hunted by the Russian nobility.^104^ In the same year as Engelhardt, Collins wrote a report on the use of deer horn as a medicament.^105^ It is interesting that the first extant import record including deer horn dates to 1673, sometime after Collins’ report, and that shipments of animal-based medicines were more common in the years after the two reports were produced, although it should be remembered that there were more recorded imports in the later period.^106^ These reports apparently represent the Apothecary Chancery reassessing the role of animal parts in medicine in the second half of the seventeenth century, a consideration which was at least partially acted upon.

As animal-based medicines could be created locally, it might perhaps be expected that animal parts would feature more heavily in the lists of medical ingredients purchased from local Russian markets than imported from overseas. At least some animal-based medicaments were considered valuable outside the court: in 1691, the Apothecary Chancery pupil Leontii Tikhanov was sentenced to be beaten for stealing a bezoar from the department, speaking to a value for that object beyond the court.^107^ Moreover, several Russian-language medical books include animal parts in their recipes.^108^ However, animal parts feature only rarely in lists of medicines the court purchased from local markets: in 1682 the Apothecary Chancery bought some ram’s horn; in 1694, they bought both spermaceti and crabs’ eyes.^109^ In fact, the department more commonly procured spices from the markets than animal parts. It is also significant that animal parts are never mentioned in any of the investigations of the medicine market trades, nor in the 1701 ban on that trade.^110^ These documents also support the idea that animal medicines were only a limited part of Russian medicine as practised.

The trajectory of the two medicines directly referred to in the Apothecary Chancery’s oath—theriac and mummia—is rather different from that of animal-based medicines as a group. Indeed, only one import document, from 1679, lists theriaca andromachi; none list mummia.^111^ The situation had changed by the early eighteenth century. In 1711, Peter the Great’s renowned spin-doctor and adviser, Stefan Iavorskii, gave a sermon entitled *Theriaca ex venenis confecta* [Theriac, made from poison], which uses theriac as a metaphor for finding success in defeat.^112^ This fits with other evidence that suggests that the ban on theriac, and possibly by extension, mummia, was defunct by the early eighteenth century. Dutch documents discussing the Russian tariff of 1731 mentions theriac as a product with a set tariff price, suggesting it was regularly traded to Russia by that date.^113^ The Russian court’s ban on theriac and mummia was certainly in effect in the 1640s–1660s, but was waning even by the late 1670s, and had apparently disappeared completely by the early eighteenth century.

Following Schiebinger’s arguments about the importance of non-transfers in the history of cross-cultural medical exchange, we can see evidence from the oaths as an important corrective for the evidence from the import lists. Although Russia drew substantially on Western European sources and practices for their medical drugs, that was always a selective process, and in the case of medical drugs, corpse medicine and animal-based medicine were substantially limited in the first half of the seventeenth century, although that restriction waned later in the century. This evidence echoes the conclusions drawn by Levin and Renner regarding other areas of early modern Russian medicine: the Russian court spent substantial time, effort and money importing Western European medical practitioners, books and practices. However, this was not a slavish imitation of Western European norms. Rather, that importation went hand-in-hand with the selection and adjustment of that medicine to suit Russian purposes.

## Conclusion

Devoting attention to documents relating to medical drugs reveals aspects of early modern Russian medicine, and indeed aspects of early modern European medicine more generally, that other sources cannot. Medical drugs in Russia tell us about the relationship between official and unofficial medicine, and revise our ideas of the relationship of Russian medicine with medicine elsewhere in Europe, and indeed the world. Histories of early modern Russian medicine have almost exclusively focused on the Apothecary Chancery, and understandably so, as the records of that institution represent a substantial collection of medical documents for that period. The patient circle of the Apothecary Chancery expanded during the course of the century, but even by the 1690s it only encompassed a minority of Russians, with the vast majority looking elsewhere for their health care. In fact, the Apothecary Chancery records provide the opportunity to examine unofficial healing practices, in particular the medical drugs stocked by local markets and purchased from them by the court. The very fact of these documents demonstrates that there was a consensus between unofficial and official medicine regarding a proportion of medical drugs. Indeed, even new medical drugs like the American imports had made it onto the markets by the 1690s. Despite the notable disjunctions, and even conflicts, between official and unofficial medicine in other areas, in the sphere of medical drugs there was at least some agreement.

This agreement covered the value not only of local drugs, but also of products from much further afield: spices from East Asia, and American products. This alters our understandings of the geographical limits of Russian medicine. Medical practitioners, and medical texts, came into Russia from a small number of Western European nations. In stark contrast, medical drugs were regularly sourced from within the empire, from East Asia, and even from the Americas. Western Europe remains an important part of the picture—both the East Asian and the American products were commonly imported via Western Europe—but it takes the role of a middleman, not a dominant actor. The continued significance of Western Europe means that Russian sources can also tell us about trends there: the fact that the Russian court could purchase such substantial amounts of both spices and American plants from Hamburg, among other places, in the middle of the seventeenth century tells us that the spice trade was of lasting importance across the century. It also shows us that American drugs were heavily traded across Europe by the middle of the century, substantially undermining claims that they were never a significant part of European medicine. The Russian court’s interest in recording its purchases reveals the state of medical drugs both within Russia, and across Europe, as being consistently geographically diverse.

Conversely, despite the continued importance of Western Europe to Russian medicine, Russian drug records do not show a slavish imitation of Western European trends. Indeed, two of the most popular and well-regarded medicines of early modern Europe—mummia and theriac—were banned from the Russian court at the start of the seventeenth century, although that ban did not last more than a few decades. Here evidence from medical drugs once again echoes other work on Russian medicine, notably the conclusions of both Eve Levin and Andreas Renner, that early modern Russian medicine was highly and fundamentally selective of Western European trends and models. This is an important point for placing Russian medicine within current discussions of global histories. Broad trends are always modified by local considerations, in this case making plants grown on a continent whose very existence was recent knowledge preferable to medicines known in Europe for centuries.

## Acknowledgements

The research upon which this paper is based was supported by grants from the Wellcome Trust, and the Max Planck Institute for the History of Medicine. I am grateful, as ever, to the staff of the Russian State Archive of Ancient Documents [RGADA], Moscow, for the use of their materials, and their patience and support. Earlier versions of this paper were presented at the workshops ‘Trading Medicines: The Global Drug Trade in Perspective’ at the London School of Economics, and ‘Continuity and Change in Russian Therapy’, at the University of Oxford. My thanks go to the participants of those workshops, as well as Rachel Koroloff, Erika Monahan, Matthew Romaniello, Patrick Wallis, two anonymous peer reviewers and my collaborators in creating this pair of articles, Jan Willem Veluwenkamp and Werner Scheltjens, for their invaluable comments on previous drafts of this article. Any remaining errors are entirely my own.

## Funding

This work was supported by the Wellcome Trust and the Max Planck Institute for the History of Science.

## Footnotes

1 After 1654 the department began training Russians as medical practitioners. The reasons for starting the training programme are unknown, but it may be significant that 1654 saw the start of the Russo-Polish war (1654–67), as well as plague outbreaks. Maria Unkovskaya, ‘Learning Foreign Mysteries: Russian Pupils of the Aptekarskii Prikaz, 1650–1700’, *Oxford Slavonic Papers*, 1997, 30, 1–20.

2 Andreas Renner, *Russische Autokratie und europäische Medizin. Organisierter Wissenstransfer in 18. Jahrhundert* (Stuttgart: Franz Steiner, 2010), 55.

3 Clare Griffin, ‘In Search of an Audience: Popular Pharmacies and the Limits of Literate Medicine in Late Seventeenth- and Early Eighteenth-Century Russia’, *Bulletin for the History of Medicine*, 2015, 89, 705–32.

4 The only works to devote attention to the drug trade are John Appleby, ‘Ivan the Terrible to Peter the Great: British Formative Influence on Russia’s Medico-Apothecary System’, *Medical History*, 1983, 27, 289–304; V. N. Shkunov, ‘Aptekarskii prikaz i vneshniaia torgovlia Rossii v XVII–nachale XVIII vv.’, *Moskovskoe nauchnoe obozrenie*, 2011, 6, 2–3; B. Z. Nanzatov and M. M. Sodnompilova, ‘Lekarstvennye sredstva v torgovo-obmennykh operatsiiakh mezhdu Rossiei, Mongoliei i Kitaem v XVII–XIX vv.’, *Vestnik nauchnogo tsentra Sibirskogo otdeleniia Rossiiskoi akademii nauk*, 2014, 4, 90–8.

5 Jarmo T. Kotilaine, *Russia’s Foreign Trade and Economic Expansion in the Seventeenth Century: Windows of the World* (Leiden: Brill, 2005), 11.

6 See Wilhelm Richter, *Geschichte der Medicin in Russland* 3 vols (Moscow: N. S. Vsevoloski, 1813–17); N. P. Zagoskin, *Vrachi i vrachebnoe delo v starinnoi Rossii* (Kazan: Tipografiia Imperatorskogo universiteta, 1891); N. Ia. Novombergskii, *Cherty vrachebnoi praktike v Moskovskoi Rusi (kultur’no-istoricheskii ocherk)* (St Petersburg: Tipografiia Ministerstva vnutrennykh del, 1904); N. Ia. Novombergskii, *Nekotorye spornye voprosy po istorii vrachebnogo dela v do-Petrovskoi Rusi* (St Petersburg: Tipografiia Ministerstva vnutrennykh del, 1903); M. B. Mirskii, *Meditsina Rossii X–XX vekov. Ocherki istorii* (Moscow: ROSSPEN, 2005); M. B. Mirskii, *Ocherki istorii meditsiny v Rossii XVI–XVIII vv.* (Vladikavkaz: Reklamno-izdatelskoe agentstvo Goskomizdata RSO-A, 1995); Unkovskaya, ‘Learning Foreign Mysteries’; Maria Unkovskaya, *Brief Lives: A Handbook of Medical Practitioners in Muscovy, 1620–1701* (London: Wellcome Trust, 1999); Sabine Dumschat, *Ausländischer Mediziner im Moskauer Russland* (Stuttgart: Franz Steiner Verlag, 2006); Eve Levin, ‘The Administration of Western Medicine in Seventeenth-Century Russia’, in Jarmo Koitlaine and Marshall Poe, eds, *Modernizing Muscovy. Reform and Social Change in Seventeenth Century Russia* (London and New York: Routledge Curzon, 2004), 353–79; M. Sokolovskii, *Kharakter i znachenie deiateĺnosti Aptekarskogo prikaza* (St Petersburg: P. P. Soikin, 1904).

7 Eve Levin, ‘Healers and Witches in Early Modern Russia’, in Yelena Mazour-Matusevich and Alexandra S. Korros, eds, *Saluting Aron Gurevich: Essays in History, Literature, and Other Related Subjects* (Leiden: Brill, 2010), 105–33.

8 V. A. Kovrigina, ‘Apteki i aptekari Moskvy vtoroi poloviny XVII—pervoi chetverti XVIII v.’ *Vestnik Moskovskogo universiteta seriia 8 Istoriia*, 1999, 1, 38–70. The decree is published in *Polnoe Sobranie Zakonov Rossiiskoi Imperii s 1649 goda* 40 vols (St Petersburg: Tipografii otdelenia sobstavennoi ego Imperatoskogo Velichestva kantseliarii, 1830), IV, 117.

9 This literature is now sufficiently large that a comprehensive citation would exceed the bounds of this article. For some assessments of the arguments over the start of globalisation, see Giorgio Riello, ‘The Globalisation of Cotton Textiles: Indian Cottons, Europe and the Atlantic World, 1600–1850’, in Riello, Giorgio, and Prasannan Parthasarathi, eds, *The Spinning World: A Global History Of Cotton Textiles, 1200–1850* (Oxford: Oxford University Press, 2011), 261–87, see 261–6; and Jan Nederveen Pieterse, ‘Periodizing Globalization: Histories of Globalization’, *New Global Studies,* 2012, 6, 1–25. For an assessment of globalisation and the drug trade, see Benjamin Breen, ‘Portugal, Early Modern Globalization and the Origins of the Global Drug Trade’, *Perspectives on Europe*, 2012, 42, 84–8.

10 Matthew P. Romaniello, ‘Through the Filter of Tobacco: The Limits of Global Trade in the Early Modern World’, *Comparative Studies in Society and History*, 2007, 49, 914–37.

11 See for example Kotilaine, *Russia's Foreign Trade*, vii.

12 Kotilaine, *Russia’s Foreign Trade*, 64.

13 Dumschat, *Ausländische Mediziner*, 101.

14 T. A. Oparina, *Inozemtsy v Rossii XVI–XVII vv.* (Moscow: Progress-Traditsiia, 2007).

15 Maria Unkovskaya cites a document confirming the existence of the *aptechnaia izba* in 1572: Unkovskaya, ‘Learning Foreign Mysteries’, 4–5; see also Levin, ‘Administration’, 365; Dumschat, *Ausländische Mediziner*; Peter B. Brown, ‘How Muscovy Governed: Seventeenth-Century Russian Central Administration’, *Russian History*, 2009, 36, 459–529, 501; John T. Alexander, ‘Medical Development in Petrine Russia’, *Canadian-American Slavic Studies*, 1974, VIII, 198–221.

16 Unkovskaya, ‘Learning Foreign Mysteries’.

17 Kovrigina, ‘Apteki i aptekari’.

18 Dumschat, *Ausländische Mediziner*, 101.

19 Such was the case for members of the defeated Naryshkin faction, the losers in a dynastic struggle, A. S. Matveev and K. P. Naryshkin, in 1677. See Levin, ‘Administration’, 362.

20 Document dated 1632 recording medicines and a field surgeon being sent to the army. Russian State Archive of Ancient Documents, Moscow [henceforth RGADA] f. 143, collection of the Apothecary Chancery, op. 1, ed. khr. 114.

21 Levin, ‘Administration’, 357.

22 Levin, ‘Administration’, 357; Unkovskaya, ‘Foreign Mysteries’, 33; N. E. Mamonov, *Materialy dlia istorii medistiny v Rossii* 4 vols (St Petersburg: Tipografiia M. M. Stasiulevich, 1881), III, 899–901.

23 For recent work on this, see Mirskii, *Meditsina Rossii X–XX vekov*; Unkovskaya, *Brief Lives*; Dumschat, *Ausländischer Mediziner*; Levin, ‘The Administration of Western Medicine’.

24 Levin, ‘Healer and Witches’, 125–7; N. Ia Novombergskii, *Materialy po istorii meditsiny v Rossii*, 5 vols (St Petersburg: M. M. Stasiulevich, 1905), I, 12–13.

25 Kovrigina, ‘Apteki i aptekari’, writes about the Moscow markets.

26 Renner, *Russische Autocratie*, 237–44.

27 Levin, ‘Healers and Witches’.

28 See for example Valerie Ann Kivelson, *Autocracy in the Provinces: The Muscovite Gentry and Political Culture in the Seventeenth Century* (Stanford, CA: Stanford University Press, 1996); Matthew Romaniello, *The Elusive Empire: Kazan and the Creation of Russia, 1552–1671* (University of Wisconsin Press, 2012); Michael Khodarkovsky, *Russia’s Steppe Frontier: The Making of A Colonial Empire, 1500–1800* (Bloomington–Indianapolis: Indiana University Press, 2002).

29 One exception is Rachel Koroloff, ‘Seeds of Exchange: Collecting For Russia’s Apothecary And Botanical Gardens in the Seventeenth and Eighteenth Centuries’, (unpublished PhD, University of Illinois at Urbana-Champaign, 2014).

30 Alix Cooper, *Inventing the Indigenous. Local Knowledge and Natural History in Early Modern Europe* (Cambridge: Cambridge University Press, 2007).

31 Mamonov, *Materialy*, II, 139–43.

32
*Ibid*., 155–7.

33 Receipts dating from 1672, Mamonov, *Materialy*, II, 470–71. 1694 receipts RGADA f. 143, op. 2, ed. khr. 1554.

34 RGADA f. 143, op. 3, ed. khr. 462.

35 Levin, ‘Healer and Witches’, 125–7; Novombergskii, *Materialy*, I, 12–13.

36 Levin, ‘Healer and Witches’.

37 Novombergskii, *Materialy*, III, part 1, 9–12.

38 Broken-wind refers to a form of allergic bronchitis that causes wheezing, coughing and laboured breathing in horses. The quote is from Mamonov, *Materialy*, III, 676–77.

39 Novombergskii, *Materialy*, I, 60–61.

40 Rachel Koroloff, *Seeds of Exchange*, 41.

41 Janet Martin, *Treasure of the Land of Darkness: The Fur Trade and its Significance for Medieval Russia* (Cambridge: Cambridge University Press, 2004).

42 Koroloff, *Seeds of Exchange*, 41.

43 Clifford M. Foust, *Rhubarb: The Wondrous Drug* (Princeton, NJ: Princeton University Press, 1992).

44 Erika Monahan, ‘Locating Rhubarb: Early Modernity’s Relevant Obscurity’, in Paula Findlen, ed., *Early Modern Things. Objects and their Histories, 1500–1800* (London and New York: Routledge, 2013), 227–51; Monahan, ‘Regulating Vice and Virtue: Controlling Commodities in Early Modern Siberia’, in Matthew Romaniello and Tricia Starks, eds, *Tobacco in Russian History and Culture. From the Seventeenth Century to the Present* (London and New York: Routledge, 2011), 61–82; Monahan, *The Merchants of Siberia. Trade in Early Modern Eurasia* (Ithaca and London: Cornell University Press, 2016), 55, 60–1, 103, 125, 173–4, 185, 192, 266–70, 274. My thanks to Erika Monahan for her thoughts on rhubarb.

45 Mamonov, *Materialy*, II, 155–7.

46 For the Arkhangelsk annual fair, see Mamonov, *Materialy*, II, 470–71; For the Moscow markets, see

47 Mamonov, *Materialy*, II, 155–7.

48 Richter, *Geschichte der Medizin*, I, 448–55.

49 Mamonov, *Materialy*, I, 79–86.

50 RGADA f. 143, op. 2, ed. khr. 1513.

51 Richter, *Geschichte der Medizin*, I, 448–55.

52 See Mamonov, *Materialy*, I, 15.

53 See for example Mamonov, *Materialy*, IV, 1071–6.

54 Mamonov, *Materialy*, I, 79–86.

55 Kotilaine, *Russia’s Foreign Trade*, 133.

56 Dumschat, *Ausländische Mediziner*, 105.

57 Harold J. Cook, and Timothy D. Walker, ‘Circulation of Medicine in the Early Modern Atlantic World’, *Social History of Medicine*, 2013, 26, 337–51, 339.

58 Patrick Wallis, ‘Exotic Drugs and English Medicine: England’s Drug Trade, *c*. 1550–*c*. 1800’, *Social History of Medicine*, 2012, 25, 20–46.

59 Mamonov, *Materialy*, II, 155–7.

60
*Ibid*., II, 228.

61 For 1651: Mamonov, *Materialy*, II, 139–43; for 1684:

62 RGADA f. 143, op. 2, ed. khr. 1554.

63 RGADA f. 143, op. 1, ed. khr. 1a.

64 Mamonov, *Materialy*, II, 334–46. For a conversion table of contemporary Russian to modern weights, see Richard Hellie, *The Economy and Material Culture of Russia, 1600–1725* (Chicago: University of Chicago Press, 1999), 646.

65 Twenty *funty* were imported in 1684, RGADA f. 143, op. 3, ed. khr. 112. In 1692 ½ *funt* was imported, RGADA f. 143 op. 2 ed. khr. 1445. In 1694 2 *funty* were imported, RGADA f. 143 op. 2 ed. khr. 1513.

66 Hellie, *Economy*, 648. In 1647, 16 *zolotniki* of cinnamon were purchased. Mamonov, *Materialy*, III, 601–4.

67 The smallest quantity for this period was 15 *funty* in 1684. RGADA f. 143, op. 3, ed. khr. 112. The largest was 100 *funty* in 1691. RGADA f. 143 op. 2 ed. khr. 1442.

68 Hellie, *Economy*, 646.

69 The 1646 shipment, Mamonov, *Materialy*, III, 601–4. The 1691 shipment, RGADA f. 143 op. 2 ed. khr. 1442.

70 Wallis, ‘Exotic Drugs’.

71 Valeria Finucci, ‘“There’s the Rub”: Searching for Sexual Remedies in the New World’, *Journal of Medieval and Early Modern Studies*, 2008, 38, 523–57, 532.

72 Teresa Huguet-Termes, ‘New World Materia Medica in Spanish Renaissance Medicine: From Scholarly Reception to Practical Impact’, *Medical History*, 2001, 45, 359–76; Wallis, ‘Exotic Drugs’.

73 Trade links with Spain and Portugal emerged only in the mid-eighteenth century. V. N. Zakharov, *Zapadnoevropeiskie kuptsy v rossiiskom torgovle XVII veka* (Moscow: Nauka, 2005), 200–9, 218–30. Following numerous voyages along the Pacific Coast of North America across the eighteenth-century, the Russian state formally took possession of areas in Alaska, Hawai’i and Southern California in the early nineteenth century. See Ilya Vinkovetsky, *Russian America: An Overseas Colony of a Continental Empire, 1804–1867* (Oxford: Oxford University Press, 2011).

74 RGADA f. 143, op. 2, ed. khr. 1554.

75 Richter, *Geschichte der medicin*, I, 448–55.

76 Huguet-Termes, ‘New World *Materia Medica*’, 368–70.

77 The 1645 shipment, Mamonov, *Materialy*, I, 79–86. 1647 shipment, Mamonov, *Materialy*, III, 601–4. The 1660 shipment, Novombergskii, *Materialy*, I, 9–10. The 1667 shipment, Mamonov, *Materialy*, II, 334–46. The 1673 shipment, Mamonov, *Materialy*, II, 517–22. The 1691 shipment, RGADA f. 143, op. 2, ed. khr. 1442. The 1692 shipment, RGADA f. 143, op. 2, ed. khr. 1445. The 1694 shipment, RGADA f. 143, op. 2, ed. khr. 1513.

78 The 1660 shipment, Novombergskii, *Materialy*, I, 9–10. The 1667 shipment, Mamonov, *Materialy*, II, 334–46. The 1694 shipment, RGADA f. 143, op. 2, ed. khr. 1513.

79 Timothy D. Walker, ‘The Medicines Trade in the Portuguese Atlantic World: Acquisition and Dissemination of Healing Knowledge from Brazil (*c*. 1580–1800)’, *Social History of Medicine*, 2013, 26, 403–31, 429; Wouter Klein, and Toine Pieters, ‘The Hidden History of a Famous Drug: Tracing the Medical and Public Acculturation of Peruvian Bark in Early Modern Western Europe (*c*. 1650–1720)’, *Journal of the History of Medicine and Allied Sciences*, 2016, 2–22.

80 The 1667 shipment, Mamonov, *Materialy*, II, 334–6. The 1694 shipment, RGADA f. 143, op. 2, ed. khr. 1513.

81 Walker, ‘Medicines Trade’, 430.

82 The 1683 shipment, RGADA f. 143, op. 3, ed. khr. 106. The 1692 shipment, RGADA f. 143, op. 2, ed. khr. 1445. Robert S. Munger, ‘Guaiacum, the Holy Wood from the New World’, *Journal of the History of Medicine*, 1949, 4, 196–229.

83 Mamonov, *Materialy*, III, 601–4.

84 Mamonov, *Materialy*, II, 334–6.

85 RGADA f. 143, op. 2, ed. khr. 1445; RGADA f. 143, op. 2, ed. khr. 1513.

86 Robert Collis, ‘Magic, Medicine and Authority in Mid-Seventeenth-Century Muscovy: Andreas Engelhardt (d. 1683) and the Role of the Western Physician at the Court of Tsar Aleksei Mikhailovich, 1656–1666’, *Russian History* 2013, 40, 399–427.

87 Finucci, ‘There's The Rub'.

88 Wallis, ‘Exotic Drugs’; Huguet-Termes, ‘New World *Materia Medica*’.

89 Levin, ‘Administration’; Renner, *Russische Autokratie*, 38–53.

90 Londa Schiebinger, *Plants and Empire*. *Colonial Bioprospecting in the Atlantic World*, (Cambridge, MA: Harvard University Press, 2009).

91 A. M. Kleimola, ‘The Duty to Denounce in Muscovite Russia’, *Slavic Review*, 1972, 31, 759–79, 764.

92 For oaths from the period of Mikhail Fedorovich, see Mamonov, Materialy, I, 48–57. For oaths from the reign of Aleksei Mikhailovich, see RGADA, f. 143, op. 3, ed. khr. 548.

93 Mamonov, *Materialy*, I, 48–57, 49.

94 Richard Sugg, *Mummies, Cannibals, and Vampires: The History of Corpse Medicine from the Renaissance to the Victorians* (Abingdon, UK and New York: Routledge, 2011).

95 Karl H. Dannenfeldt, ‘Egyptian Mummia: The Sixteenth Century Experience and Debate’, *Sixteenth Century Journal*, 1985, 16, 163–80; Nancy G. Siraisi, *History, Medicine, and the Traditions of Renaissance Learning* (Ann Arbor: University of Michigan Press, 2007), 229–30.

96 Christiane Nockels Fabbri, ‘Treating Medieval Plague: The Wonderful Virtues of Theriac’, *Early Science and Medicine*, 2007, 12, 247–83.

97 Sugg, *Mummies, Cannibals, and Vampires*, 23.

98 Marshall Poe, ed., *Early exploration of Russia: The Present State of Russia: In a Letter to a Friend at London* (London: Routledge-Curzon, 2003), 20.

99 Poe, *Early Exploration of Russia*, 19.

100 RGADA f. 143, op. 2, ed. khr. 749.

101 Richter, *Geschichte der Medizin*, I, 448–55; Mamonov, *Materialy*, I, 79–86.

102 RGADA f. 143, op. 2, ed. khr. 1442.

103 Novombergskii, *Materialy*, I, 54–5.

104 Ann Kleimola, ‘Hunting for Dogs in 17th-Century Muscovy’, *Kritika: Explorations in Russian and Eurasian History*, 2010, 11, 467–88.

105 RGADA f. 143, op. 2, ed. khr. 734.

106 Mamonov, *Materialy*, II, 517–22.

107 RGADA f. 143, op. 2, ed. khr. 1432.

108 See for example N. S. Kotkova, ‘Lechebnik poslednei treti XVII v.’, in V. G. Dem’ianov and N. I. Tarabasov, eds, *Istochniki po istorii russkogo iazyka XI-XVII vv* V. G. Dem’ianov and N. I. Tarabasov (Moscow: Otdelenie rukopisei IRIA RAN, 1991), 173–95.

109 A 1682 purchase of ram’s horn, RGADA f. 143, op. 3, ed. khr. 42. A 1694 purchase of spermaceti and crabs’ eyes, RGADA f. 143, op. 2, ed. khr. 1554.

110 Case dating from 1679, Mamonov, *Materialy*, IV, 1110–11. Case from 1682, Mamonov, *Materialy*, IV, pp. 1288–89. Case from 1699, RGADA f. 143, op. 3, ed. khr. 462. Decree dated 1701, *Polnoe Sobranie Zakonov*, IV, 117.

111 Mamonov, *Materialy*, IV, 1122–5.

112 Robert Collis, *The Petrine Instauration: Religion, Esotericism and Science at the Court of Peter the Great, 1689–1725* (Leiden: Brill, 2011), 268. My thanks to Robert Collis for pointing me towards this issue.

113 Stadsarchief Amsterdam, collection no. 78, Archief van de Directie van de Oostersche Handel en Reederijen: document 415.

